# Assessing environmental health impacts of coal mining exploitation in Iran: A Rapid Impact Assessment Matrix (RIAM) approach for environmental protection

**DOI:** 10.1371/journal.pone.0293973

**Published:** 2023-12-07

**Authors:** Wang Tianliang, Zahra Aghalari, Raphael Mubanga, Juan Eduardo Sosa-Hernandez, Manuel Martínez-Ruiz, Roberto Parra-Saldívar

**Affiliations:** 1 School of Management, Changchun University, Changchun, China; 2 Faculty of Public Health, Babol University of Medical Sciences, Babol, Iran (I.R. Iran); 3 Environmental Planning and Management Japan International Research Centre for Agricultural Sciences-JIRCAS, Tsukuba, Ibaraki, Japan; 4 Tecnologico de Monterrey, School of Engineering and Sciences, Campus Monterrey, Monterrey, Nuevo León, Mexico; Xi’an University of Science and Technology, CHINA

## Abstract

Environmental Impact Assessment is the process of evaluating the effects caused by a project on the environment. The outcomes generated by this assessment can lead to a reduction of the negative effects and an increase in the positive effects caused by mine projects. The present study was conducted to evaluate the environmental impact assessment of the Goliran Coal Mine in northern Iran. In the descriptive-analytical study, to achieve the objectives, observatory surveys were conducted around the coal mine using a checklist, which was about the positive and negative effects of a coal mine. Then the data were entered into the RIAM and the positive and negative effects were ranked and the most important effects were determined. In RIAM, one point is assigned to each component. 17 important activities for environmental impacts were identified using a checklist. Among the activities carried out at the coal mine site, the major ones included tunnel excavation, construction of the rail line collection and disposal of coal mine effluent, coal transportation, collection and disposal of mine tailings, and technical defects and leakage. The scores of each environmental factor were based on the four environmental components: physical/chemical, biological/ecological, social/cultural, and economic/operational. The results of the present study showed that the most negatively affected environmental components are the physical/chemical components derived from three activities; the construction of the underground tunnel; the construction of a coal transport rail line; and the actual transportation of coal extracts. The scores of each environmental factor based on the four components at the Goliran coal mine in northern Iran indicate that the highest negative score was -64, corresponding to the physical/chemical component, and was assigned to air pollution. On the other hand, the highest positive score corresponds to the economic/operational component with +54, assigned to the income that employees earn from the mine. Overall results showed that the coal mine in northern Iran had negative effects on the environment but the effects were not severe. It is suggested that for future research, corrective measures should be taken in the form of an environmental management plan to reduce the negative effects caused by coal mining, and then prospective research should be done to check the extent of reducing the negative effects.

## 1. Introduction

Coal is a non-renewable fossil fuel formed from plant remains under high pressure and heat over millions of years. Coal mining creates wealth and job opportunities in different regions. However, it has negative effects on the environment i.e., water and air pollution, occupational hazards due to exposure of workers to pollution, high risk of developing a disease in the near residents, changes in the traditional values of a society, and biodiversity loss [1, 2]. In a study by Kayet et al., (2022) it was shown that the vegetation around the mine is being destroyed due to human activities in the coal mine [3]. In a study by Singh et al., (2023) it was shown that soil pollution was one of the most important negative environmental effects of coal mines [4]. In a study by Mithal Jiskani et al., (2023) it was shown that mining can cause air and water pollution, but safe and smart methods such as Green and climate smart mining (GCSM) are used to reduce the negative effects of mining on the environment [5]. In a study by Mithal Hendryx et al., (2020) it was shown that coal mining in Australia has caused environmental pollution, especially air pollution and particulate emissions [6].

Environmental protection is essential for the welfare and health of the international community and sustainable economic development. Today, the way to achieve sustainable development is to pay attention to industries and mines that are profitable and practice principles of resource removal, while preserving the environmental principles. This approach will improve people’s lives in society but not pose a serious threat to future generations [7, 8]. For this reason, most countries in the world weigh the effects of the construction of industrial projects on the health of society and the environment before the final decision on industrial and mining projects [9]. Environmental Impact Assessment (EIA) is a tool designed to identify and predict the consequences of a project on the environment, health, hygiene, and well-being of communities. EIA began in the United States of America (USA) as early as the 1970s. Since that decade, more than 100 countries have adopted EIA and have so far formulated EIA policies, legislation, and institutions to implement this tool [10–12].

Preparing an environmental impact assessment report is essential for industry and mining projects as well as other development projects [7]. One way to inform people about the adverse effects of industrial and mining projects is to assess the environmental impacts and show society how they can minimize the negative effects and increase the positives [13]. For example, in a study by Mancini et al., (2018) it was shown that the evaluation of the effects of the construction and operation of mines can strengthen positive effects such as the development of economic and business in the region [14]. In a study by Emmanuel et al., (2018) it was shown that the environmental impact assessment of mines can be used to determine the environmental and health effects of mines, such as water pollution, forest destruction, reduction of soil nutrients, destruction of wildlife habitat, and threats to human health [15].

### 1.1. Environmental impacts associated with coal mining methods

Coal mining can be done by two methods, open-pit and underground, where each one has a list of environmental impacts. First, open-pit mining involves digging for coal near the ground surface. Open-pit mining destroys trees, plants, soil, forests, and wildlife habitats. Moreover, this type of mining leads to loose soil and soil erosion [16, 17]. The rain carries loose soil to rivers, also it is also susceptible to a mudslide. The soil that enters into the river affects the life under the water and also blocks water canals, leading to floods in the area. Drilling, blasting, and heavy vehicle operations in mines cause noise pollution and vibrations. In addition, mining drilling affects the water quality of rivers due to the contamination of water by toxic gases, heavy metals from extraction operations, and changes in the pH of the water. Changes in water quality affect agricultural and livestock activities [18].

Most coal is mined underground. Although underground mining is less destructive than open-pit mining, it has destructive effects. When soil and rock wastes from mines come in contact with air and water, they cause pollution [18, 19]. Open and underground mining reduces water levels and harms the access of people to groundwater such as wells [20]. In addition, mines produce methane, which is a greenhouse gas. The use of coal as a fossil fuel releases methane which also leads to climate change. The Pegasus Foundation reports that coal mines produce 6% of methane [20].

### 1.2. Existing environmental impact evaluation methods in EIA

There are several methods to assess the environmental impact of activities and projects (see summary in Table 1). These methods include checklists, matrices, and networks [21–23]. Canter in 1996 claimed that EIA is a complex method and there are simpler methods for assessing environmental impacts [24]. Below is a description of the methods.

**Table 1:** Main advantages and disadvantages of impact identification methods [25, 26].

Method	Advantages	Disadvantages
Checklists	Easy to understand and use Good for site selection and priority setting Simple ranking and weighting	Do not distinguish between direct and indirect impacts Do not link action and impact The process of incorporating values can be controversial
Matrices	Link action to impact A good method for displaying EIA results	Difficult to distinguish direct and indirect impacts Have the potential for double-counting impacts
Networks	Link action to impact Useful in simplified form for checking for second-order impacts Handles direct and indirect impacts	Can become very complex if it’s used beyond the simplified version
Overlays	Easy to understand Focus and display spatial impacts Good siting tool	Can be cumbersome Poorly suited to address impact duration or probability

**Checklists**: These are the first and simplest methods used in EIA. Checklists are used to show the expected effects. Also, they are used to indicate expected impacts. Types of checklists include simple, descriptive, and multi-attribute tool checklists [25, 26].

**Matrices**: Matrices are used to show the effects of projects on environmental factors. Using matrices is possible to identify the initial effects and make a comparative analysis of the environment. The most common matrices are the Leopold matrix and the Rapid Impact Assessment (RIAM) [25, 26].

**Networks**: Networks are used to represent primary, secondary, and tertiary development effects in projects. The purpose of using networks is to combine the causes and consequences of environmental impacts with the construction of projects. One of the most commonly used networks is the Sorensen network [25, 26].

The environment is affected by construction projects in multiple ways. Depending on the construction and operational activities, some environmental factors are more affected than others. Therefore, due to the nature of the project (coal mining that continues for a long time) and the purpose of this study, we chose RIAM as the most appropriate method for this research for the following reasons:

- The mining project consists of extensive and cumulative work which are not accountable to checklists and other matrices. Explicitly, it does not consider spatial and temporal project activity effects [27].
- RIAM has a high ability for analysis, reproducibility, high accuracy, and flexibility [28, 29]
- RIAM combines quantitative and qualitative aspects of environmental assessment (positive and negative impacts) using standard assessment criteria and rating scales according to current and future operations [29, 30].
- RIAM is more complex than checklists but simpler than networks and coverages.

### 1.3. Environmental impact assessment in Iran

In Iran, inappropriate policies and misuse of natural resources led to environmental degradation. This situation led the government to adopt the EIA for some large industrial and commercial projects to protect the environment and comply with the Sustainable Development Goals [16, 28]. The legal basis for EIA in Iran is the 2^nd^ National Development Plan (NDP) of 1994–1998. NDPs are five-year programs drafted by the Iranian government. Hence EIA was introduced in 1994 under Note 82 of the 2^nd^ NDP. This Note stated that “EIA reports should be provided during the feasibility and site-selection studies for any large projects”. The 5^th^ NDP of 2010–2015 did not only focus on conducting EIA but also mentioned the Strategic Environmental Assessment (SEA) of plans and programs at both national and regional levels [31]. That is why in Iran large projects are subject to EIA before they are implemented.

### 1.4. Previous EIA evaluations in Iran

Several scholars have conducted EIAs in Iran on various projects using EIA matrices such as the Leopold and RIAM. For example, a study was conducted in Tiam Biston steel mills and the results showed that the most negative environmental effects were in the construction phase in group (-A) and the operation phase in category (-B). The most important positive environmental effects of the Tiam Biston steel mills were on the economic income [32]. Another study conducted at a landfill in Semnan showed that the negative effects of construction activities on the environment were low. Air and soil pollution was the most important environmental problem in landfills. The positive effects of building a landfill in Semnan were the creation of green spaces, employment, and increased income [28]. A similar study on the effects of landfills in Zanjan was conducted using the RIAM matrix, which showed that the highest negative impact (-D) was related to the negative effects on animals, agriculture, and health in the region [33]. Again with RIAM, an assessment was conducted for landfills in Ghaemshahr comparing Leopold and RIAM matrices. The results showed that the construction of a sanitary landfill has the least negative impact on the environment and is the most desirable management option [34].

### 1.5. The necessity of the current study

Iran has forests, diverse vegetation, a diversity of animal species, seas, and northern and southern coasts. Several studies [35–38] indicate that significant changes as a result of overdevelopment, unsustainable development, and population growth have had adverse effects on the environment in Iran. As a result, Iran faces serious environmental challenges, including water scarcity, deforestation and desertification, habitat destruction, wetland death, soil erosion, and climate pollution. If these problems are not controlled, they can cause an environmental catastrophe. If the health and environmental authorities do not pay enough attention to the environment in Iran, the country will face a crisis that is a threat to the lives of humans, animals, and plants [35].

The adverse effects of the construction and operation of coal mines on the population of animals, and plants and environmental pollution in a country like Iran can cause climate changes and changes in the flora and fauna in other countries and the world. Therefore, this study was conducted to evaluate the environmental impact of the Goliran coal mine in northern Iran using the RIAM method to identify the environmental parameters of the coal mine. Assessing the environmental impact of the coal mine provides useful information for informed decision-makers and project managers.

According to the mentioned contents, the objectives of this study include:

1- Investigating the environmental effects of the Goliran coal mine
2- Identifying the positive effects of the construction and operation of the Goliran coal mine
3- Identifying the negative effects of the construction and operation of the Goliran coal mine

The questions of this study include:

1- What are the environmental effects of the Goliran coal mine?
2- What are the positive effects of the construction and operation of the Goliran coal mine?
3- What are the negative effects of the construction and operation of the Goliran coal mine?

## 2. Materials and methods

### 2.1. Methodology

This is a descriptive-analytical study. In this research, the positive and negative effects of the construction and operation of the Goliran coal mine were identified using a checklist. Then the information in the checklist was entered into the RIAM and the data was analyzed. The RIAM analysis was developed by Christopher Pastakia [16]. The advantages of using RIAM are in-depth analysis, high accuracy, flexibility, and the ability to perform an objective assessment [16]. A study by Mondal et al. (2010) used RIAM to evaluate the environmental effects of the urban solid waste landfill site, and the results showed that the superiority of the RIAM method over other methods is in the transparency and permanence of the analysis process [39]. In a study by Kuitunen et al. (2008) it was shown that comparing the results of environmental impact assessment (EIA) and strategic environmental assessment (SEA) using the RIAM rapid assessment matrix, the results showed that the RIAM method can be used to compare and rank individual projects, plans, programs [40]. According to the materials mentioned about the advantages of RIAM, in this study, this method was used to evaluate the environmental effects of coal mining.

RIAM is based on the definition of evaluation criteria, which allows the collection of semi-quantitative values for each criterion and provides an accurate score for each condition based on the values. The effects of project activities on environmental factors are assessed. In RIAM, one point is assigned to each component. During environmental impact assessment with RIAM, environmental components are grouped into four categories in rows and criteria in the matrix column. The criteria are grouped into two categories:

(A) Relevant criteria for the condition can individually influence the score obtained.
(B) Valuable criteria for each situation that should not individually be capable of changing the score obtained.

The value ascribed to each group of criteria is determined using a series of equations. The scoring system requires a simple multiplication of each criterion score given to the group (A). Using the coefficient is important for group (A) because it represents the weight of each score. A simple sum of scores can provide the same results for different situations. Criteria scores (B) are added together to form a single sum. The sum of the group (B) is multiplied by the results of the group (A) scores to obtain the final evaluation score (ES). The RIAM process is presented in Eqs 1–3 as follows:

$$\left({\text{A}}_{1}\right)*\left({\text{A}}_{2}\right)={\text{A}}_{\text{T}},$$ (1)

$$\left({\text{B}}_{1}\right)+\left({\text{B}}_{2}\right)+\left({\text{B}}_{3}\right)={\text{B}}_{\text{T}},$$ (2)

$$\left({\text{A}}_{\text{T}}\right)*\left({\text{B}}_{\text{T}}\right)=\text{ES,}$$ (3)

where,

(A_1_) and (A_2_) are the individual scores for the group (A);

(B_1_), (B_2_), and (B_3_) are the individual scores for the group (B);

A_T_ is the result of multiplication of all (A) scores;

B_T_ is the result of the summation of all (B) scores;

ES is the environmental score for the condition.

### 2.2. Assessment criteria

The judgment on each component is made by the criteria and scales shown in Table 2. In Table 2, the numerical scale according to the effects is presented by Pastakia et al. (1998) [16].

**Table 2:** Assessment criteria [16].

Criterion	Score	Description
A_1_	4	Important to national/international interests
Importance of condition	3	Important to regional/national interests
	2	Important to areas immediately outside the local condition
	1	Important only to the local condition
	0	No importance
A_2_	+3	Major positive benefit
The magnitude of change/effect	+2	Significant improvement in the status quo
	+1	Improvement in the status quo
	0	No change to the status quo
	-1	Negative change to the status quo
	-2	Significant negative change
	-3	Major dis-benefit or change
B_1_	1	No change
Stability	2	Temporary
	3	Permanent
B_2_	1	No change
Reversibility	2	Reversible
	3	Irreversible
B_3_	1	No change
Cumulative	2	Non-cumulative/single
	3	Cumulative/synergistic

To use the evaluation system described above, for each project option, a matrix design is needed. The matrix contains cells that represent the criteria used against each component defined. Inside the cell, individual criteria scores are defined, and ES is calculated and recorded from the Eqs (1), (2) and (3). After calculating the ES, the ES points are placed in the Range Band (RB) to provide a more accurate system for measurement (see Table 3).

**Table 3:** Environmental classification according to RIAM [16].

Environmental Score (ES)	Alphabetic (Range value)	Numeric (Range value)	Description of range band (change/impact)
72 to 108	E+	5	Major positive
36 to 71	D+	4	Significant positive
19 to 35	C+	3	Moderate positive
10 to 18	B+	2	Positive
1 to 9	A+	1	Slight positive
0	N	0	No change/status quo/not applicable
-1 to -9	A-	-1	Slight negative
-10 to -18	-B	-2	Negative
-19 to -35	-C	-3	Moderate negative
-36 to -71	-D	-4	Significant negative
-72 to -108	-E	-5	Major negative

### 2.3. Environmental components

In the RIAM method, the factors were divided into the following four general categories, each of which has more detailed factors:

- Physical/chemical (PC): noise pollution, air pollution, water pollution, soil pollution, etc.
- Biological/Ecological (BE): plants, animals and habitats, etc.
- Sociological/Cultural (SC): population, migration, traffic, health and education indicators, welfare, etc.
- Economic/Operational (EO): employment, income from coal, land prices, etc.

### 2.4. Study area background information

This descriptive-analytical study was conducted to evaluate the environmental impact of the Goliran coal mine, which was established in 1993. The Goliran coal mine was about 2500 square kilometers in 60 kilometers of the Galogah in the Bandpey region of Babol in northern Iran.

## 3. Results

### 3.1. The results of the area survey

According to the informants, the coal extracted from the Goliran mine in Northern Iran is one of the highest quality coal in the country. The initial coal production is estimated to be around 50,000 tons per year. At the time of this study, the mine had more than 100 employees. At the mine site, wastewater from the workers’ residence was discharged through an absorber well. The method of coal extraction at the Goliran mine was by underground mining due to the location of the coal layers. Hence to access these coal layers, an underground tunnel was excavated. This type of extraction had advantages such as low mining costs, high production efficiency, and low labor requirements. However, it also had some disadvantages, such as high investment costs and the possibility of leakage.

### 3.2. Scoping and the RIAM analysis

We conducted the scoping analysis and we identified 17 activities using a checklist. Among the 17 activities carried out at the coal mine site, the major ones include tunnel excavation, rail line construction for collection and disposal of coal mine effluent, coal transportation, collection and disposal of mine tailings, and technical defects and leakages. The scores of each environmental factor based on the four components at the Goliran coal mine in northern Iran indicate that the highest negative score was -64, corresponding to the physical/chemical component and was assigned to air pollution. On the other hand, the highest positive score corresponds to the economic/operational component with +54, assigned to the income that employees earn from the mine (Table 4). However, some activities did not have an environmental impact due to small magnitude or are less important. Despite having positive effects as well, we were unable to conduct a Cost-Benefit Analysis because doing so would have compromised the negative impacts. A Cost-Benefit Analysis would give us the wrong impression that no mitigation measures are needed. Indicating that both, the negative and positive impacts, allow the project implementers to reduce the negative impacts while enhancing the positive ones.

**Table 4:** Scores of each environmental factors affected by the Goliran coal mine.

Environmental factors	Criteria	A_1_	A_2_	B_1_	B_2_	B_3_	ES	RV
Physical/chemical (PC) components	Noise pollution	2	-2	2	3	2	-28	-C
	Air pollution	4	-2	3	2	3	-64	-D
	Dust	2	-3	2	2	3	-42	-D
	Surface water pollution	3	-2	3	2	3	-48	-D
	Groundwater pollution	3	-2	2	2	3	-42	-D
	Soil pollution	3	-1	3	2	3	-24	-C
	Earth shape	1	-2	3	2	2	-14	-B
	Slip and drift	1	-1	2	2	1	-5	-A
	Microclimate change	1	-3	3	2	1	-18	-B
Biological/ecological (BE) components	Animal migration	2	-2	2	3	1	-24	-C
	Animal population	2	-2	2	2	1	-20	-C
	Animal habitat	2	-2	2	3	1	-24	-C
	Plant habitat	1	-2	2	3	1	-12	-B
	Plant density	2	-2	1	2	1	-16	-B
	Food chains	2	-1	2	3	1	-12	-B
	Variety of plant species	1	-1	1	2	1	-4	-A
	Animal species diversity	2	-1	1	2	1	-8	-A
Social/cultural (SC) components	Population	1	1	1	1	1	3	+A
	Migration	1	0	1	1	1	0	N
	Traffic	1	-1	3	3	1	-7	-A
	Social acceptance	1	1	1	1	1	3	+A
	Health indicators	1	0	2	1	1	0	N
	Educational indicators	1	0	1	1	1	0	N
	Old buildings	1	-1	3	2	1	-6	-A
	Welfare	1	1	1	1	1	3	+A
	The exploitation of the mine	3	3	2	2	1	45	+D
	Land use	1	3	3	2	1	18	+B
Economic/operational (EO) components	Employment	2	3	3	2	1	36	+D
	Mine income	3	3	3	2	1	54	+D
	Prices of land around the mine	1	1	2	3	1	6	+A

Environmental factors used for the EIA were also determined based on the 17 activities carried out at the coal mine site. These components include 9 physical/chemical (PC); 8 biological/ecological (BE); 10 sociological/cultural; and 3 economic/operational components (EO). The results of the components and the scoring are shown in Table 5.

**Table 5:** Summary of the scores.

Range	-108	-71	-35	-18	-9	0	1	10	19	36	72
	-72	-36	-19	-10	-1	0	9	18	35	71	108
Class	-E	-D	-C	-B	-A	N	A	B	C	D	E
PC	0	4	2	2	1	0	0	0	0	0	0
BE	0	0	3	3	2	0	0	0	0	0	0
SC	0	0	0	0	2	3	3	1	0	1	0
EO	0	0	0	0	0	0	1	0	0	2	0
Total	0	4	5	5	5	3	4	1	0	3	0

**Fig 1:**
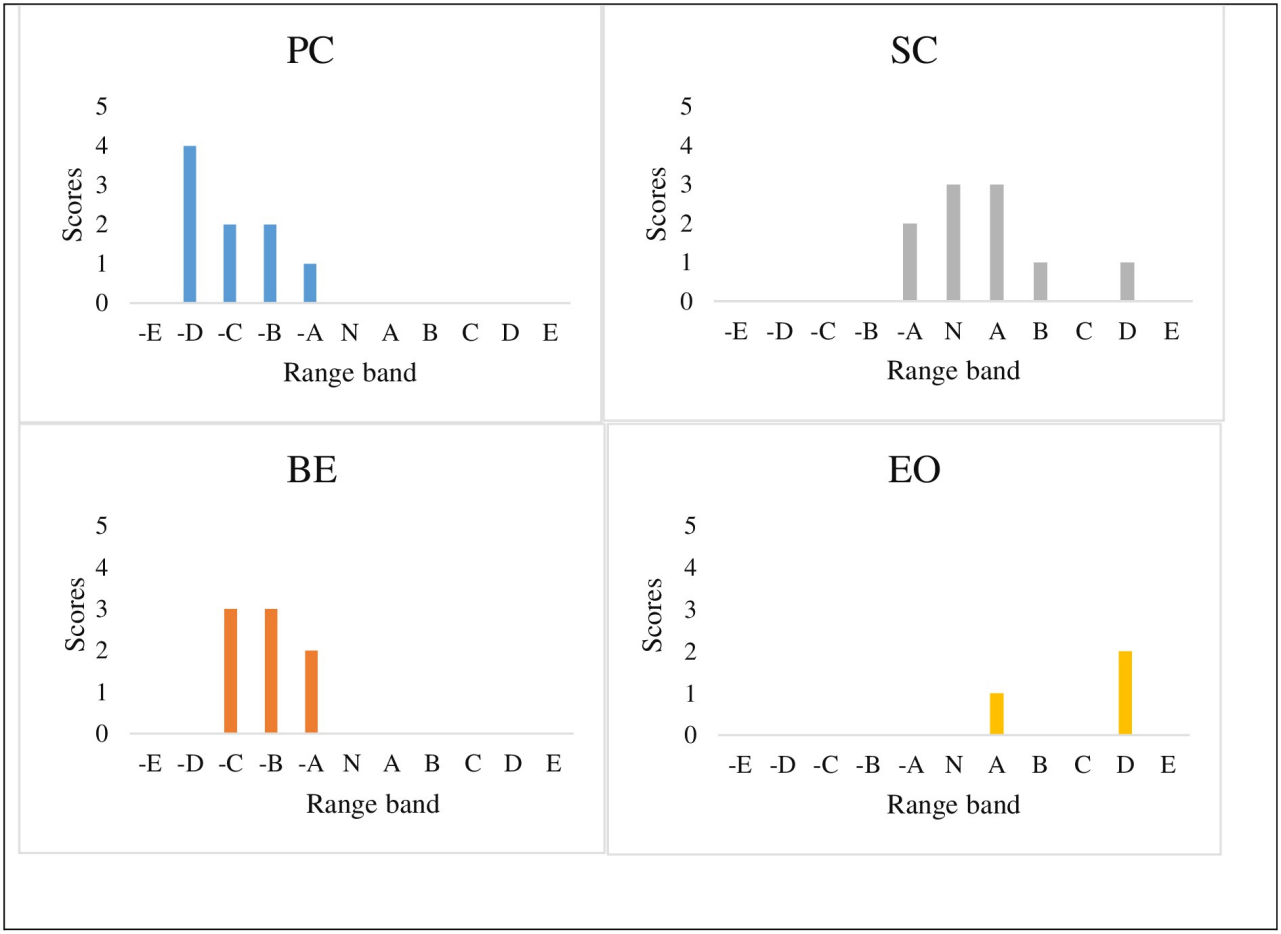
Graphical representation of the RIAM results corresponding to the four components of environmental factors.

**Fig 2:**
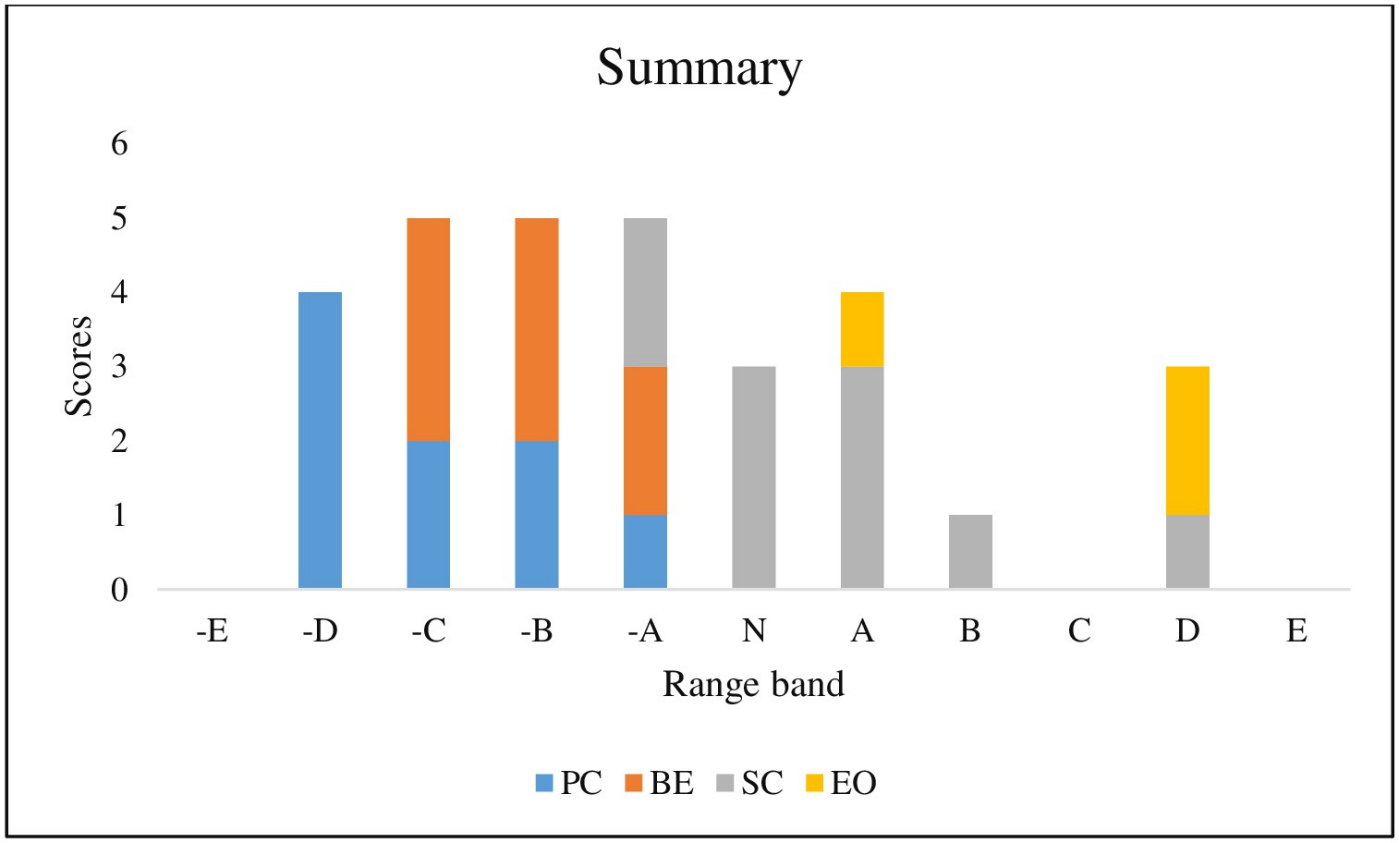
Graphical representation of the summary scores.

The range bands are indicated on the X-axis of the histogram while the scores are indicated on the Y-axis (Figs 1 & 2).

The N indicates neutral, which means no severe impacts. On the left side to N indicate the negative impacts of various components. As greater the distance from N to the components on the left, the more negative the impacts are. On the other hand, as greater the distance from N to the components in the right, the more positive the impacts are. Therefore, the more positive the impacts, the more benefits the people obtain from the project.

## 4. Discussion

The results of this study indicate that the RIAM was successfully used to evaluate the impact of the Goliran coal mine in Northern Iran. The method proved to be transparent because it indicates clearly where the scores are coming from by indicating the environmental components that were affected. Other methods can also be used for environmental impact assessment. In a study by Chen et al., (2023) it was shown that the grey analytic hierarchy process and grey clustering method are used to evaluate the construction level of green mines [41]. In a study by Jiskani et al., (2022) it was shown that the risk assessment model in MATLAB Fuzzy Toolbox is useful for evaluating the effects of mine [42].

The results of this study indicate that the most negatively affected environmental components correspond to the physical/chemical components (air pollution, dust, and surface and groundwater pollution), with a score of -D as a result of tunnel excavation and construction of the coal transport rail line. Followed by the biological/ecological components (animal migration, animal population, and animal habitat), with a score of -C due to constructions and coal transportation activities. This means that the environmental components more negatively affected, in this case -D and -C, call for the most urgent attention and intervention. The results also confirm the observations of the Environment South Africa, SGU [19, 43] who asserted that underground mining also exerts overwhelming damage to the environment in terms of air and water pollution and the collapse of old buildings due to blasts. Although most of the studies that have been conducted in Iran such as those reported by Abedinzadeh (2013) [28], Monzavi (2015) [33], Madani (2016) [32], and Asadi Shirin & Gholamalifard (2015) [34] who involve landfills and not mining, their findings are similar to our studies results because mining also involves the disposal of waste effluent. Consistently with our study, scores from most of the previous evaluations do not reach -E, which means that the negative impacts do not reach the extremes. However, project implementers should try all the alternatives to reduce these impacts further and enhance the positive ones.

On the other hand, the most positively affected components fell under the EO components (local employment and mine income), with a score of +D, followed by the sociological/cultural components (exploitation of the mine and improved land use), with a score of +B. Most of the components with an N score correspond to sociological/cultural components because there was no significant displacement of the residents; that’s why no migration or change in the educational indicators was noticed. Therefore, the results confirm Ogbonna’s views [44] that although coal mining brings wealth to communities, it also destroys the environment. In a study by Paltasingh et al., (2023) [45] it was shown that coal mining brings economic benefits, but it can endanger the health of the environment, humans, and society [45]. In a study by De Valck et al., (2021) it was shown that environmental protection has more economic effects than coal mining because in the long term, coal mining can destroy the climate of a region and destroy local communities [46].

## 5. Conclusion and recommendations

The results of this study showed that the most negative effects of the construction and operation of the Goliran coal mine were related to the physical/chemical components caused by the construction of the underground tunnel, the construction of the coal transportation rail line, and the transportation of coal extracts and these caused noise, air and water pollution. Other effects of the Goliran coal mine on the environment include the destruction of plants and animal habitats. Hence animals had to migrate to other areas. Therefore, we recommend that the management at Goliran coal mine should urgently pay attention to these components by devising mitigation measures.

The measures that can be taken to reduce the destructive effects of coal mines are as follows:

- Use machines equipped with dust control systems to prevent air pollution.
- Changing the method of coal extraction and using wet and hydraulic extraction methods.
- Measurement of pollutants from the mine such as dust particles and chemicals on a seasonal and annual basis.
- Preventing the distribution of waste from coal extraction in the environment and using the appropriate waste disposal method.
- Restoration of vegetation that was destroyed during the construction and productivity of the coal mine.
- The presence of an environmental expert and the use of his/her suggestions to prevent environmental degradation.

It is necessary to consider environmental considerations for mining activities to achieve sustainable development and prevent environmental pollution, so it is suggested to study the following in future research:

- Evaluation of the positive and negative environmental effects of mines before and after construction and operation.
- Comparison of the environmental effects of the construction and operation of coal mines in different climatic regions, hot, dry, cold, and humid.
- Evaluation of the environmental effects of mining construction through several methods in a comparative manner.
- Evaluation of the environmental effects of the construction of mines from the point of people of the region.
- Sampling of soil, water, and plants around the mines.
