# Expression of Concern: Assessing environmental health impacts of coal mining exploitation in Iran: A Rapid Impact Assessment Matrix (RIAM) approach for environmental protection

**DOI:** 10.1371/journal.pone.0358719

**Published:** 2026-09-24

**Authors:** The PLOS One Editors

The *PLOS One* Editors issue an Expression of Concern for this article [1] to notify readers of concerns about the article’s compliance with the PLOS Authorship policy.

Readers are advised to interpret the article [1] with caution.
